# Microalgae as a Source of Valuable Phenolic Compounds and Carotenoids

**DOI:** 10.3390/molecules27248852

**Published:** 2022-12-13

**Authors:** Jan Cichoński, Grzegorz Chrzanowski

**Affiliations:** 1 Doctoral School, University of Rzeszow, St. Rejtana 16C, 35-959 Rzeszow, Poland; 2 Department of Biotechnology, Institute of Biology and Biotechnology, University of Rzeszow, Zelwerowicza 8B, 35-601 Rzeszow, Poland

**Keywords:** phenolic compounds, carotenoids, microalgae

## Abstract

Microalgae are photosynthetic, eukaryotic organisms that are widely used in the industry as cell factories to produce valuable substances, such as fatty acids (polyunsaturated fatty acids (PUFAs) eicosapentaenoic acid (EPA) and docosahexaenoic acid (DHA)), sterols (sitosterol), recombinant therapeutic proteins, carbohydrates, vitamins, phenolic compounds (gallic acid, quercetin), and pigments (β-carotene, astaxanthin, lutein). Phenolic compounds and carotenoids, including those extracted from microalgae, possess beneficial bioactivities such as antioxidant capacity, antimicrobial and immunomodulatory activities, and direct health-promoting effects, which may alleviate oxidative stress and age-related diseases, including cardiovascular diseases or diabetes. The production of valuable microalgal metabolites can be modified by using abiotic stressors, such as light, salinity, nutrient availability, and xenobiotics (for instance, phytohormones).

## 1. Introduction

Microalgae are microscopic, unicellular, photosynthetic organisms which may be prokaryotic or eukaryotic, that have developed pigment-rich double membrane-bound plastids, which accumulate chlorophylls and accessory pigments, mainly carotenoids [1,2]. These organisms belong to algae, which, along with the land plants, are a part of the green lineage (*Viridiplantae*) that originated from the ancient endosymbiotic event, in which cyanobacterium was captured by the eukaryotic cell and eventually turned into a plastid. According to the proposed hypothesis, this lineage diverged from a green flagellate into two clades. The first one was *Streptophyta,* which is a clade that land plants belong to, along with their ancestors, charophyte green algae. The other line was *Chlorophyta,* which includes the majority of described green algae species [3]. Green algae are vastly diversified in terms of their morphology and ecology. They can reside in terrestrial, freshwater, and marine habitats, where they are abundant in marine phytoplankton. Their size varies, as green algae range from microalgae to macroalgae [4]. The wide diversification of algae posed the need for the development of molecular tools capable of distinguishing between species. Currently, algae can be classified by comparing their 18S rRNA/rDNA, *rbcL,* or ITS2 sequences and relating them to the documented databases [5,6,7]. Scientists around the world have extensively studied green algae, especially microalgae, and their enormous productivity. Since the early 2000s, the market demand for high-value products and algae cultivation locations has flourished [8]. These organisms have been reported to fix 1.83 kg of atmospheric carbon dioxide per 1 kg of algal biomass and convert it to useful carbon-based products, making algae not only efficient producers but also greenhouse gas reducers, which may be a potential solution to global warming. They produce nearly half of Earth’s oxygen and can fix 100 million tons of carbon dioxide per day [2,9,10]. Microalgae were extensively used to produce commercial products as early as the 1940s, with the proposal of exploiting them for the extraction of lipids. Today, industries isolate from microalgae such valuable compounds as fatty acids (such as polyunsaturated fatty acids (PUFAs) eicosapentaenoic acid (EPA) and docosahexaenoic acid (DHA), which can prevent many diseases, including cardiovascular diseases or cancer), sterols (such as sitosterol), recombinant therapeutic proteins, carbohydrates, vitamins, phenolic compounds (for instance, flavonoids), and pigments (such as carotenoids β-carotene and astaxanthin) [9,11,12]. 

Green algae are advantageous to higher plants for the production of bioactive substances because they grow faster, have high biomass productivity, fix high volumes of carbon dioxide, and utilize small amounts of water [10]. On the other hand, they can be used to generate green energy carriers, such as bioethanol, biogas, and biohydrogen [13]. With their astounding capacities, bioactive microalgal compounds can be used to produce pharmaceuticals or nutraceuticals for therapeutic purposes, animal feed, and even food colorants and additives for the food industry [14]. As some authors suggest, there may be a way to produce unconventional bioactive phenolic substances, such as pharmacologically active cannabinoids [15], or antiviral agents that can possibly be used against SARS-CoV-2, such as phycocyanobilins, lectins, and sulfated polysaccharides [16]. In this brief review, we illustrate the enormous value of the bioactive compounds that are produced by green algae. Carotenoids and phenolic compounds, including flavonoids and phenolic acids, are potent substances that are valuable to many industries and human health. Creating new and optimized microalgal culture processes, with knowledge of substances and stressors that can improve the productivity of those incredible organisms, can lead to the progress of algal biotechnology. Hereafter, we briefly characterize these two groups of compounds, demonstrate their capabilities, and describe the current production of carotenoids and phenolic compounds. In addition, we show that abiotic stress conditions can lead to the overproduction of valuable compounds.

## 2. Bioactive Metabolites Produced by Microalgae and Their Beneficial Effects

### 2.1. Phenolic Compounds

Phenolic compounds are an abundant class of secondary metabolites used in various physiological processes in plants, including the stress response, allowing the organism to interact and adapt to its surroundings and survive harsh conditions, such as UV radiation. Phenolic compounds are expressed by higher plant organs, such as fruits, vegetables, and spices. Moreover, algae are natural sources of these molecules. Several classes of flavonoids, such as flavonols, flavanones, isoflavones, and dihydrochalcones, were found in microalgae and cyanobacteria. Furthermore, phenolic acids, such as gallic, protocatechuic, caffeic, chlorogenic, vanillic, *p*-hydroxybenzoic, and salicylic acids, were found in different species of marine algae [17,18]. The concentration of algal polyphenols within algal cells varies according to the season, habitat, and factors such as UV irradiation, light, nutrient availability, and salinity [19]. Phenolic compounds have two basic chemical structures. The first one is a C6-C1 ring structure, which is a basic skeleton of phenolic acids and hydrolyzed tannins. Phenolic acids are the simplest and most important nonflavonoid compounds. They contain a single phenyl group that is substituted for a carboxylic and one or more -OH groups. Phenolic acids are classified by the length of the chain that contains the carboxylic group and, upon that, are split into hydroxybenzoic acids and hydroxycinnamic acids; classification is based on the length of the chain that contains the carboxylic group. Another structure is the C6-C3-C6 ring, shared by phenylpropanoid acids, flavonoids, and condensed tannins [20,21,22]. Flavonoids are the most abundant phenolic compounds that contain a phenyl benzopyran skeleton, with two phenyl rings bonded through a heterocyclic pyran ring. Based on the differences in the bond between benzene and pyran ring and by substitution patterns to the pyran ring, they are divided into several main subgroups, including flavonols, flavones, flavanones, flavanols, isoflavonoids, and anthocyanins [23]. The biosynthesis of phenolic compounds is carried out through the metabolism of phenylpropanoids in the presence of precursors from the shikimic and malonic acid pathways [22,24].

Bulut et al. [25] have extracted antioxidant substances from microalga *Scenedesmus* sp. using different solvents. The total phenolic compounds concentration was the highest in the ethanol/water (3:1 *v*/*v*) extract, which amounts to 5.40 ± 0.28 mg GAE/g DW, and in the ethyl acetate extract (3.73 ± 0.65 mg GAE/g DW). RP-HPLC analysis revealed that the highest amount within phenolic compounds was attributed to the flavonoid quercetin in ethyl acetate extract 844.5 ± 125.0 μg/g DW, whereas its content in ethanol/water extract was 551.9 ± 90.9 μg/g DW. Additionally, gallic acid (653.6 ± 54.3 μg GAE/g DW), 4-hydroxy benzoic acid (441.9 ± 30.0 μg GAE/g DW), and chlorogenic acid (352.0 ± 45.0 μg GAE/g DW) were abundant in the ethanol/water extract. Andriopoulos et al. [26] have demonstrated that from five tested microalgal species, *Dunaliella. salina* at the late stationary phase had the highest total phenolic content in the aqueous extract (8.78 ± 1.49 mg GAE/g DW), whereas it reached 1.30 ± 0.37 mg GAE/g DW within the methanolic extract. The highest phenolic content within the methanolic extracts was reported in *Chlorella minutissima* at the early stationary phase (9.04 ± 0.68 mg GAE/g DW), while the phenolic content in its aqueous extract only reached 3.00 ± 0.30 mg GAE/g DW. Bhuvana et al. [27] have identified in HPLC chromatogram bands representing chicoric acid and caffeic acid derivative, and in *Nannochloropsis oculata*, the highest peaks were attributed to quinic acid derivative, quercetin pentosidehexoside, and luteolin 7-*O*-glucoside.

#### 2.1.1. Biosynthesis of Phenolic Compounds

**Figure 1:**
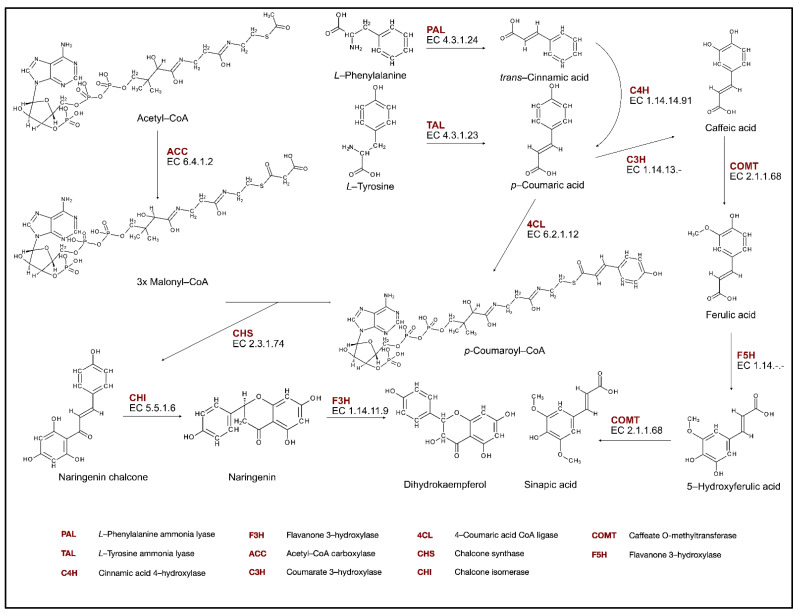
Phenolic compounds biosynthesis pathway.

Flavonoids are synthesized by the phenylpropanoids and polyketide pathways. First, the shikimate pathway provides aromatic amino acids, phenylalanine, and tyrosine, which are precursors to initiate the phenylpropanoid pathway (PP). The first enzyme of this pathway, which leads to the production of lignins, lignans, and flavonoids, is phenylalanine ammonia lyase (PAL, EC. 4.3.1.24) (Figure 1). This enzyme catalyzes the formation of *trans*-cinnamic acid from phenylalanine, with the associated loss of ammonia. Then, *trans*-cinnamic acid is hydroxylated in position C-4 by the cytochrome P450 monooxygenase—cinnamic acid 4-hydroxylase (C4H, EC. 1.14.14.91). This yields *p*-coumaric acid, which is then activated to the corresponding CoA thioester. The reaction is catalyzed by 4-coumarate-CoA ligase (4CL, EC. 6.2.1.12) [28].

The synthesis of specific flavonoids begins with the formation of naringenin chalcone, which is derived from *p*-coumaroyl-CoA and three molecules of malonyl-CoA, with the activity of chalcone synthase (CHS). Malonyl-CoA originates from acetyl-CoA in the reaction catalyzed by acetyl-CoA carboxylase. Chalcone is the first key intermediate in the flavonoid biosynthesis pathway (FBP) because it provides a basic skeleton for downstream flavonoid synthesis. The second key enzyme in the FBP is chalcone isomerase (CHI), which catalyzes the intramolecular cyclization of chalcones to form flavanones in the cytoplasm and the heterocyclic ring C in the flavonoid biosynthesis pathway [29]. 

**Figure 2:**
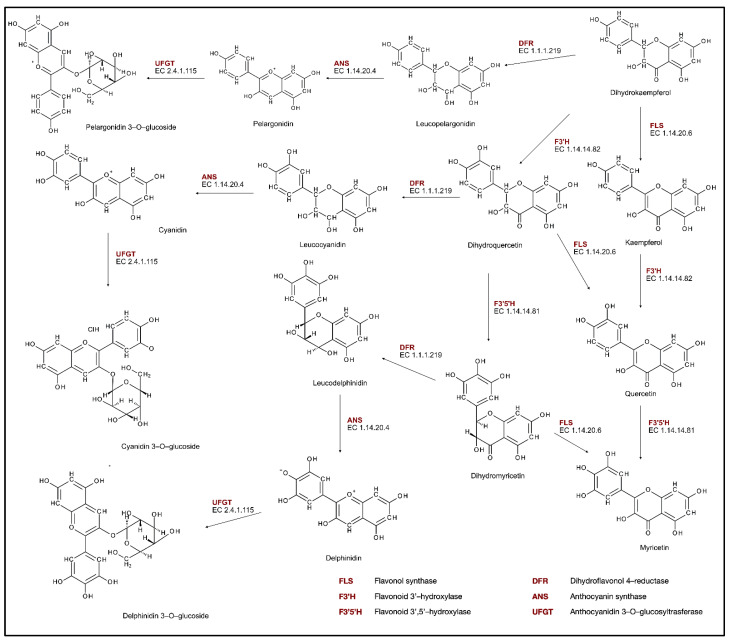
Phenolic compounds biosynthesis pathway.

After the flavanone naringenin is produced, it can be oxidized by flavanone 3−hydroxylase (F3H, EC. 1.14.11.9) to form dihydrokaempferol (Figure 2), which can be hydroxylated by flavonoid 3′monooxygenase (F3′H, EC. 1.14.14.82) on the 3′ position of the B-ring, or flavonoid 3′,5′-hydroxylase (F3′5′H, EC. 1.14.14.81) on 5′ position, which produces correspondingly dihydroquercetin or dihydromyrcetin. Dihydroflavonols are then converted to unstable anthocyanidins by the reduction and oxidation reactions catalyzed by dihydroflavonol reductase (DFR, EC. 1.1.1.219) and anthocyanin synthase (ANS, EC. 1.14.20.4). The formed cyanidin, pelargonidin, and delphinidin are then used in the glycosylation reaction catalyzed by anthocyanidin 3-*O*-glucosyltransferase (UFGT, EC. 2.4.1.115) to produce anthocyanins [30,31].

Although phenolic acids and flavonoids are present in microalgae, further studies regarding phenolics within these microorganisms are needed. According to Del Mondo et al. [32], the biosynthesis of phenolic compounds in microalgae needs to be further investigated, as there is a gap in its understanding compared to the well-studied phenolic biosynthesis within higher plants. Nevertheless, Del Mondo [33] have conducted a novel in silico analysis, where they have analyzed the homologs of the plants’ enzymes across microalgae; they have shown that the core enzymes of the phenolic compounds biosynthesis pathway are conserved across algal divisions. However, the occurrence of PAL (TAL) varied between algae, and this enzyme could be partly lost in Cyanobacteria and Chlorophyta, which makes the initiation mechanism of the phenylpropanoid pathway yet unknown.

#### 2.1.2. Phenolic Compounds and Their Influence on Health

Flavonoids have numerous beneficial health effects due to their antioxidant and chelating capacities. The antioxidant activity of flavonoids in vitro is connected to the arrangement of functional groups around their nuclear structure, as the structure-activity relationship research suggests [34]. Phenolic compounds have beneficial traits that can be used in different industries, including cosmetic, drug, and food production. Their antioxidant, anti-inflammatory, antimicrobial and cytotoxic bioactivities are highly suggested [20]. Goiris et al. [35] have demonstrated that extracts obtained from microalgae possess high antioxidant capacities. In their study, they have shown that along with carotenoids, phenolic compounds contributed significantly to the antioxidant capacity of tested microalgal samples. According to Alzaabi et al. [36], the dietary intake of flavonoids can be important in the prevention and treatment of SARS-CoV-2. Several in vitro studies have shown that quercetin, herbacetin, rhoifolin, and pectolinarin can inhibit the SARS-CoV main protease activity (Mpro), which is required for the replication of the virus. Furthermore, quercetin operating in synergistic action with vitamin C may act as an anti-SARS-CoV-2 and immunomodulatory agent. The immunomodulatory and anti-inflammatory properties of flavonoids can be used to extinguish the life-threatening cytokine storm characterized in severe cases of COVID-19. Khaerunnisa et al. [37] and Tallei et al. [38] have shown, with in silico molecular docking studies, that naringenin kaempferol, quercetin, luteolin-7-glucoside, demethoxycurcumin, curcumin, apigenin-7 glucoside, oleuropein, catechin, epigallocatechin gallate, rhoifolin, and hesperidin can block COVID-19 Mpro and S-protein, thus in effect the viral infection. Li et al. [39] have demonstrated that phenolic compounds extracted from Tartary buckwheat bran inhibited the growth of human liver cancer cells (HepG2). Authors Ferdous and Balia Yusof [40] have shown the high potential of algal-derived flavonoids in relation to cancer cells. They point out that quercetin, which can be found in algae, has strong anti-oxidative activity, even more prominent than other phenolics such as rutin or catechol. In their review, they also mention that quercetin can upregulate p53, which increases p21 protein, and in effect, arrest the cell cycle of cancer cells. Moreover, it can decrease tumor metastasis. Phenolic compounds derived from algae, due to their antioxidant properties, could also be used in the treatment of Alzheimer’s disease. It is particularly important, considering that one of the suggested causes of Alzheimer’s disease is an oxidative burst and oxidative damage accumulation caused by reactive oxygen species. Furthermore, those algal-derived compounds can inhibit acetylcholinesterase and butylcholinesterase activities, which is suggested as a form of management of this neurodegenerative disease [41]. Wang et al. [42] suggest that the ethyl acetate fraction of 70% ethanol extract (SPEE) of *Spirogyra* sp., a freshwater green alga, has a protective effect on keratocytes (HaCaT cells) subjected to the UVB irradiation. This fraction reduced the apoptosis of HaCaT cells and scavenged UVB-induced ROS, where higher extract concentration resulted in higher antioxidant capacity. Moreover, the ethyl acetate fraction protected against UVB-induced oxidative stress in the in vivo zebrafish model. The online HPLC-ABTS analysis of SPEE has shown that the peaks having higher antiradical activities were identified as gallic acid, methyl gallate, and ethyl gallate (a newly isolated compound from *Spirogyra* sp.). Their research shows that gallic acid and its derivatives may be used in the treatment of UVB-induced damage to the skin. Gallic acid extracted from *Spirogyra* sp. also possess vasorelaxant and antihypertensive effects. Kang et al. [43] have shown that gallic acid extracted from *Spirogyra* sp. increased the levels of nitric oxide in a concentration-dependent manner in human umbilical vein endothelial cells (HUVECs). The authors point out that nitric oxide mediates endothelium-dependent relaxation, so it may be concluded that gallic acid has vasorelaxant capability. Moreover, gallic acid at lower concentrations than 50 μg/mL showed no cytotoxicity to the cells. Furthermore, they have demonstrated that gallic acid prevents hypertension by regulating blood pressure levels. They have proven it by claiming the decrease of blood pressure in spontaneously hypertensive rats (in vivo) and in vitro inhibition of the angiotensin-I converting enzyme (ACE), which increases blood pressure. Wali et al. [44] have found that extract from green microalga *Nannochloropsis oculata*, which contains a high amount of polyphenolic constituents (437 ± 0.95 μg QE/g of flavonoid content and 53.92 ± 0.83 mg GAE/g of phenolic content), inhibited the proliferation of MDA-MB-231 human breast cancer cell line in concentration and time-dependent manner. In this study, they also show high antimicrobial activity and the antioxidant potential of the extract. The authors, referring to the previous studies, point out that the antioxidant activity of the extract is attributed to polyphenols, carotenoids, and other compounds such as fatty acids. Furthermore, Gabr et al. [45] have demonstrated that phenolic compounds extracted from *Spirulina platensis* may be used against oxidative stress and liver injury in diabetic rats. The treatment with the extracts has decreased the levels of oxidative stress marker malondialdehyde and increased the activities of SOD, GPx, and CAT, the antioxidant enzymes. Moreover, the levels of IL-6 and TNF-α decreased, which suggests the anti-inflammatory effect of the extract. The HPLC analysis revealed that the main phenolic compounds in the extract were pyrogallol (638.50 mg/100 g), E-Vanillic acid (31.53 mg/100 g), and ellagic acid (19.83 mg/100 g), and the main flavonoid compound was hesperidin (9.01 mg/100 g). 

### 2.2. Carotenoids

**Figure 3:**
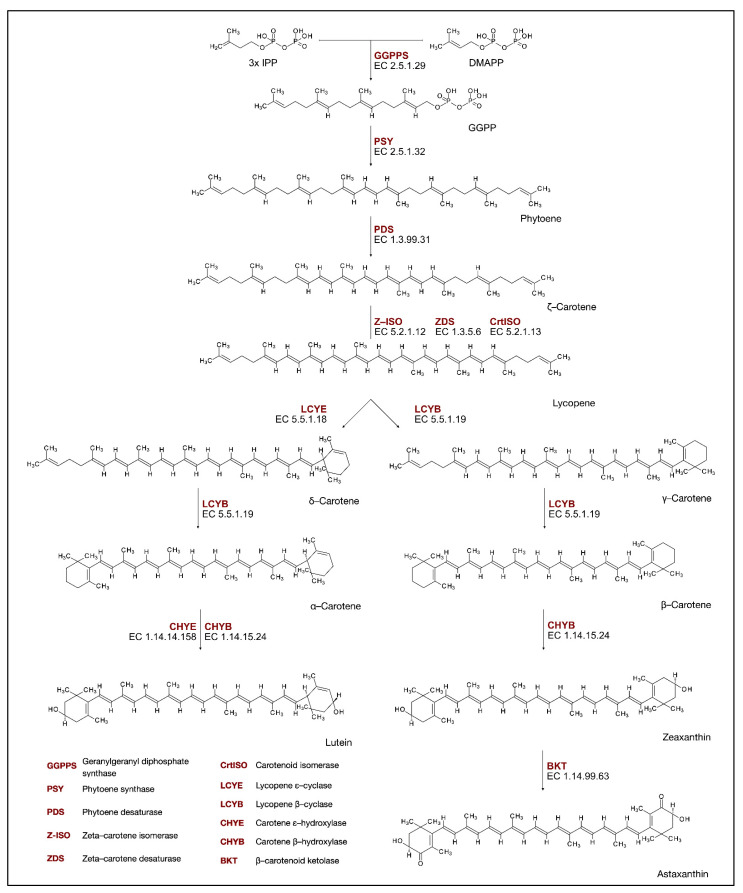
Carotenoids biosynthesis pathway.

Carotenoids are a widely distributed natural-occurring group of pigments. These lipophilic isoprenoid molecules are present in plants, photosynthetic bacteria, various species of archaea, fungi, animals, and algae. They are yellow, orange, red, and purple tetraterpene pigments. Their main structure is a forty-carbon polyene chain, with nine conjugated double bonds and an end group present at both ends. This core is made up of eight isoprene units bonded in a head-to-tail pattern. Carotenoids are divided into two distinct groups. The first group embraces hydrocarbon carotenes (Figure 3), such as α-carotene, β-carotene, lycopene, and the second, xanthophylls (Figure 3), which are carotenoids that contain an oxygen atom that forms the hydroxy, carbonyl, aldehyde, carboxylic, epoxide, and furanoxide groups. These molecules protect the photosynthetic apparatus from excess light or UV radiation in photosynthetic organisms. They are intermediates in the biosynthesis of abscisic acid and other apocarotenoids, which are the oxidative and enzymatic cleavage products of carotenoids, composed of skeletons that have fewer than 40 carbon atoms. Furthermore, carotenoids are suggested to play a signaling function between plants and their environment [46,47].

As the demand for carotenoids worldwide is soaring, a large portion of these valuable pigments is obtained from biotechnological sources, such as microalgae. Some of the most significant algal carotenoids are β-carotene, astaxanthin, canthaxanthin, and lutein (Figure 3). Microalgal production is expensive, so efforts are being made to produce new and more cost-effective mass cultivation systems. Open systems for microalgal production may cover areas ranging from several hundred to a few thousand square meters. However, their growth conditions are not optimal for several algal strains. On the contrary, closed systems are more suitable for producing most microalgae strains. They are more expensive to build and operate, but working conditions such as medium composition, temperature, pH, aeration, stirring, and irradiance can be controlled. Moreover, the risk of contamination is substantially lower [48]. Fernandes et al. [49], using HPLC-PDA-MS/MS, have found 11 carotenoids in *C. sorokiniana*, with the highest amounts of all-*trans*-lutein at 831.18 ± 1.18 μg/g DW, all-*trans*-β-carotene (156.21 ± 0.22 μg/g DW), and all-*trans*-α-carotene (71.47 ± 0.10 μg/g DW). The concentration of total carotenoids was 1408.46 μg/g. In *Scenedesmus bijuga,* the total carotenoid levels reached 1195.75 μg/g, with 16 carotenoids quantified. Additionally, in this case, the highest amount was attributed to all-*trans*-lutein at 526.40 ± 4.40 μg/g DW and all-*trans*-β-carotene (165.95 ± 1.38 μg/g DW), and unlike in *C. sorokiniana*, 9-*cis*-neoxanthin (151.52 ± 1.26 μg/g DW). Di Lena et al. [50] have identified and quantified the carotenoids in five microalgae using RP-HPLC-PDA, and they have recorded the following total levels of carotenoids: *Porphiridium cruentum*—167.16 ± 0.30 mg/100 g DW; *Isochrysis galbana*—1760.21 ± 20.84 mg/100 g DW; *Phaeodactylum tricornutum*—1022.21 ± 64.83 mg/100 g DW; *Tetraselmis suecica*—297.24 ± 10.22 mg/100 g DW; and *Nannochloropsis gaditana*—446.77 ± 41.88 mg/100 g DW. In two microalgae with the highest total carotenoids levels, the authors have identified (all-*E*)-Fucoxanthin as a carotenoid with the highest concentration in *I. galbana* (1346.49 ± 33.08 mg/100 g DW) and in *P. tricornutum* (776.84 ± 56.62 mg/100 g DW). In addition, another carotenoid with high concentration—(all-*E)*-Violaxanthin—was identified in *N. gaditana* (336.72 ± 40.50 mg/100 g DW). Moreover, β-carotene was identified in all of the tested microalgal species, with the highest level in *N gaditana* (100.06 ± 1.38 mg/100 g DW).

#### 2.2.1. Biosynthesis of Carotenoids

Isoprenoids are derived from the common precursor isopentenyl diphosphate (IPP). This molecule is synthesized by two pathways: the mevalonate pathway (MVA), which produces precursors for cytosolic and mitochondrial isoprenoids, and the plastid methylerythritol phosphate pathway (MEP). Such compartmentation of MVA in the cytoplasm and MEP in plastids has optimized isoprenoid biosynthesis in plants. Only the MEP pathway is present in green algae; therefore, these organisms have developed systems to export MEP precursors from plastids, such as IPP, so that cytosolic isoprenoids can be formed. However, higher plants and some algae have retained both pathways [51]. The carotenoids biosynthesis pathway (CBP) in algae is common to well-studied carotenogenesis in land plants but with several algae-specific pathways. Some of the genes in the CBP of algae are suggested by the homology of known genes, with reports of functional enzymes, mainly in *Chlorophyceae* [52]. A head-to-tail condensation of three IPP groups into dimethylallyl pyrophosphate (DMAPP) allyl head produces 20-carbon geranylgeranyl diphosphate (GGPP) (Figure 3). The reaction is catalyzed by geranylgeranyl diphosphate synthase (GGPPS, EC. 2.5.1.29), and the produced GGPP is used as a precursor for the biosynthesis of carotenoids [53]. During the first and well-known carotenoid biosynthesis step, a reaction catalyzed by the phytoene synthase enzyme (PSY, EC. 2.5.1.32), phytoene is synthesized from GGPP [54]. The next steps that lead to the production of lycopene from phytoene are a series of desaturation and isomerization reactions catalyzed by phytoene desaturase (PDS, EC. 1.3.99.31), zetta-carotene isomerase (Z-ISO, EC. 5.2.1.12), zetta-carotene desaturase (ZDS, EC. 1.3.5.6), and carotenoid isomerase (CrtISO, EC. 5.2.1.13) [55]. *Trans*-lycopene undergoes cyclization at one or both ends of its 40-carbon chain. Two different enzymes catalyze the cyclization reaction, as the biosynthesis pathway is split into two branches at this point. In the first, β-cyclase (LCYB, EC. 5.5.1.19), an enzyme responsible for the production of β rings, catalyzes the production of γ-carotene. In the next step, catalyzed by LCYB, β-carotene is formed. In another biosynthetic branch, ε-cyclase (LCYE, EC. 5.5.1.18) catalyzes the production of ε-ring and forms δ-carotene. Subsequently, LCYB influences the assembly of the β ring in the newly formed carotenoid, producing α-carotene, which contains both ε and β rings. These two forms of end rings diverge from each other by the double bond position [54]. The produced carotenes—α- and β-carotene—can be further hydroxylated to produce xanthophylls. β-carotene hydroxylation is catalyzed by the β-carotene hydroxylase (CHYB, EC. 1.14.15.24) in zeaxanthin production. On the other hand, α-carotene is hydroxylated by two cytochrome P450 monooxygenases (carotene ε and β hydroxylases), which produce lutein [56]. Zeaxanthin is then converted by β-carotenoid ketolase (BKT, EC. 1.14.99.63) to astaxanthin. Research suggests that in alga *Chromochloris zofingiensis,* the synthesis of astaxanthin origins from the ketolation of zeaxanthin via the intermediate adonixanthin, but the exact molecular mechanism of astaxanthin synthesis may differ between species [57].

#### 2.2.2. Effect of Carotenoids on Health

Carotenoids possess many health-promoting effects. β-carotene has been shown to be a potential anti-obesity agent. This compound is a natural precursor to apocarotenoids, including retinoids such as vitamin A. Bonet et al. [58] have shown in their review that vitamin A can modulate the function and development of adipose tissue in rodents, correlated with decreased adipogenic and lipogenic potential of adipose tissues (lower PPARγ, a transcription factor responsible from controlling adipocyte differentiation and metabolism) and increased thermogenic potential in brown adipose tissue (increased expression of UCP1 and UCP2—UCP1 can uncouple fuel oxidation from ATP synthesis and generate heat). To study the adiposity process, Amengual et al. [59] have used (*Bcmo1*^−1^) knockout mice supplemented with β-carotene, as Bcmo1 is the main enzyme in retinoid production that converts β-carotene into all-*trans*-retinal. They have observed that β-carotene supplementation in mice can provide Bcmo1-dependent downregulation of PPARγ activity in adipocytes and, in effect, control body adiposity. They point out that β-carotene intake may act differently on adiposity, depending on the Bcmo1 functional gene variant. Mukherjee and Yun [60] have shown that 3T3-L1 preadipocytes treated with β-carotene can stimulate browning in white adipocytes, where it has upregulated specific genes and proteins of beige and brown adipose tissue, including SIRT3 and SIRT6 sirtuins. UCP1, the thermogenic protein, and the fat browning marker proteins PRDM16 and PGC-1α were also upregulated. Additionally, they have shown elevated expressions of PPARγ and C/EBPα (another adipogenic transcription factor). However, lipogenesis was alleviated, suggesting that β-carotene promotes lipid catabolism in white adipocytes. 

Furthermore, carotenoids can be used as agents protecting against type 2 diabetes. Sluis et al. [61] have conducted a cohort study on the intake of dietary carotenoids and the risk of type 2 diabetes in the Dutch population. The authors have adjusted the study for lifestyle factors, such as physical activity and fiber intake. Their results have shown that diets high in β-carotene and α-carotene are related to the decreased probability of type 2 diabetes occurrence in men and women. Furthermore, Qi and Kim [62] have searched for the inhibitors of α-glucosidase, which is a main enzyme that hydrolyzes starch and disaccharides. Inhibition of this enzyme delays the digestion of carbohydrates and suppresses hyperglycemia which is characteristic of diabetes. To achieve their goals, they have purified lutein and two zeaxanthin stereoisomers from the green alga *Chlorella ellipsoidea*. They have demonstrated that the *trans* form of lutein and the *cis* form of zeaxanthin have significantly inhibited α-glucosidase activity. This may suggest that carotenoids can be used in parallel to the treatment and prevention of type 2 diabetes. Moreover, reactive oxygen species are excessively produced in diabetes due to hypoglycemia, which causes the worsening of the condition and the emergence of its complications, including cardiovascular disease (CVD), which is associated with high mortality worldwide, especially during the COVID-19 pandemic, as the UK study reports [63,64]. Cardiovascular disease can appear as an effect of oxidative stress, which is a state of failed redox homeostasis when the oxidative balance between reactive oxygen species and the antioxidant defense system is disturbed. Overproduced oxidants can cause damage to macromolecules, including protein carbonylation, lipid peroxidation, and DNA oxidation, resulting in 8-hydroxy-2′-deoxyguanosine. Furthermore, this state was associated with the presence of other pathologies, such as atherosclerosis, hypertension, cancer, Alzheimer’s disease, and, discussed earlier, diabetes [65]. Carotenoids derived from higher plants, and especially those extracted from marine sources, are attracting attention around the world. They exhibit antioxidant properties, which are attributed to their ability to quench singlet oxygen and scavenge free radicals. This may contribute to their beneficial effects on human health, including protection against inflammatory diseases, such as CVD [66]. An example here is atherosclerosis, which is a disease in which plaque buildup is present and is affected by decreased blood flow in the arteries. Its progression is related to pro-inflammatory cytokines and ROS, which oxidize low-density lipoprotein, which then accumulates in the subendothelium [67]. Two population studies have found lower levels of lutein and zeaxanthin in the serum of patients with early atherosclerosis. It may suggest that these carotenoids can prevent further development of this disease [68,69]. Algal β-carotene has shown protective effects against atherosclerosis in mice and humans, inhibiting the oxidation of low-density lipoprotein (LDL) [70]. During CVDs, when the myocardium is obstructed and the ischemic heart undergoes blood restoration treatment, a myocardial ischemia-reperfusion injury occurs. In the state of hypoxia, cardiac metabolism is altered, and reperfusion that suddenly restores cellular balance can cause injury and generate a ROS burst that can cause damage and myocardial apoptosis [71]. Xu et al. [72] have tested the influence of lycopene on myocardial ischemia-reperfusion injury (MIRI) in isolated primary neonatal cardiomyocytes from mice. MIRI can occur when the myocardium is obstructed during CVD and is subject to blood restoration treatment, which may generate ROS burst and damage. Their results demonstrate that lycopene decreased apoptosis of cells in reoxygenated cells and decreased ROS generation. Furthermore, lycopene lessened the stress in the endoplasmic reticulum (ER), which is a crucial part of the progression of ischemia-reperfusion injury. An example of decreased ER stress is the upregulation of phosphorylated AMPK, which, when inactivated, contributes to ER stress and myocardial apoptosis. Moreover, studies have shown that carotenoids supplemented in dietary doses can alleviate the risk of cancer development. Largely, the oxidative stress modulatory capacities of those compounds may influence their anticancerogenic potential. Nevertheless, several studies have shown an increased probability of cancer occurrence, especially when providing high-dose supplementation, and the increased probability of developing cancer is apparent during the β-carotene supplementation in the smoking population [73,74]. However, the study conducted by Darendelioğlu [75] illustrates that β-carotene at high concentration has a prooxidative effect on human neuroblastoma cells SH-SY5Y. The carotenoid treatment has increased ROS and lipid peroxidation in cancer cells, as well as upregulated the cas-3 expression, which concluded in cell apoptosis. Rocha et al. [76] have demonstrated that lycopene plays a hepatoprotective role. It has decreased the levels of oxidative damage in homogenized mice hepatic tissue, specifically lipid peroxidation and protein carbonyl content, and has reduced the TNF-α, IL-6, and IL-10 inflammatory cytokines. The authors Sugawara et al. [77] show that fucoxanthin and siphonoxanthin extracted from marine alga *Undaria pinnatifida* (brown macroalga) and *Codium fragile* (green macroalga) inhibit mRNA expression of FGF-2 and its receptor FGFR-1, and further the expression of FGF-2 protein in human umbilical vein endothelial cells. As FGF is an angiogenic factor, the authors have shown that algal fucoxanthin and siphonoxanthin have anti-angiogenic activities. Furthermore, Sun et al. [78] confirmed that fucoxanthin extracted from marine microalgae has the potent ability to inhibit the formation of advanced glycation end products (AGEs), which can contribute to the occurrence of chronic and age-related diseases. They have shown that the antiglycation effect of the aqueous acetone extracts from tested marine microalgae (*Phaeodactylum tricornutum*, *Cyclotella cryptica*, *Isochrysis galbana*, *Nitzschia laevis*) contained fucoxanthin as a major carotenoid. On top of that, the analyses have shown that the antiglycation effect of the extracts was positively correlated with the concentration of fucoxanthin. Additionally, the fucoxanthin standard at 60 μM inhibited the formation of AGEs in the two models by more than 60%. To summarize, fucoxanthin extracted from marine microalgae can be used as an antiglycation agent that could potentially prevent cardiovascular diseases, Alzheimer’s disease, or diabetes complications. Havas et al. [79] also demonstrated that the carotenoids present in the extracts from microalga *Dunaliella salina* inhibit the accumulation of advanced glycation end products and show anti-inflammatory activity in skin explants. The obtained hydrophobic microalgal extract contained colorless phytoene and phytofluene and colored β-carotene, although after the purification of the extract, β-carotene was not detected, leaving only colorless carotenoids within the extract. The analyses have shown that the 0.5% extract significantly lowered the levels of AGE and inflammatory interleukins 6 and 8 ex vivo. Moreover, the 1% extract antiglycation and anti-inflammatory bioactivities were confirmed during the clinical tests. The extract also possessed a potent antiaging activity. El-Baz et al. [80] have demonstrated that another compound extracted from *D. salina*, a zeaxanthin ester, zeaxanthin heneicosylate can protect against cardiac dysfunction associated with aging induced herein by *D*-galactose in tested rats. This carotenoid increased the cardiac GLUT-4 levels, a marker decreased by cardiac dysfunction, and decreased the IL-6 and iNOS levels. Furthermore, it has normalized the superoxide dismutase levels and decreased NF-κB concentration (its inhibition may prevent cardiac hypertrophy and its dysfunction). It also upregulated RAR-α gene expression, which is downregulated during cardiac dysfunction, as the authors describe. Moreover, it normalized the ECG pattern and heart rate. The authors conclude that this tested zeaxanthin ester can be a natural agent for mitigating cardiac dysfunction by the activation of retinoid receptors in cardiac tissue. A study conducted by Lin et al. [81] shows that astaxanthin extracted from *Haematococcus pluvialis* has a neuroprotective effect against the oxidative stress of optic nerve ischemia. Aside from the antioxidative activity, this carotenoid also provides anti-inflammatory and anti-apoptotic activities. It decreased the levels of IL-1β and TNFα in the retina and reduced macrophage infiltration. Moreover, it also maintained the levels of phosphorylated mTOR and its downstream factors. It promoted Akt activation and lowered the levels of cleaved caspase 3. That proves the anti-apoptotic activity of astaxanthin, which protects retinal ganglion cells from cell death. In conclusion, astaxanthin can preserve visual function and retinal cells after optic nerve ischemia. Jayappriyan et al. [82] have demonstrated that β-carotene extracted from *D. salina* inhibited the growth of the human prostate cancer cells (PC-3) more efficiently than the synthetic β-carotene.

Carotenoids are localized in three main sites of algal chloroplasts, similar to higher plants. β-carotene, small amounts of α-carotene, and the xanthophylls lutein, neoxanthin, zeaxanthin, violaxanthin, and antheraxanthin are localized in thylakoids. In chloroplast envelopes, mainly violaxanthin and lutein are present. However, trace amounts of carotenoids can also be found in plastoglobuli, which are lipoproteins surrounded by a membrane lipid monolayer and contain specialized hydrophobic metabolites and proteins that may have enzymatic functions, for example, CCD4—a carotenoid metabolic enzyme. They can accumulate several carotenoids, including lutein, lycopene, zeaxanthin, antheraxanthin, violaxanthin, and neoxanthin [83,84]. The demand for natural pigments to replace their synthetic counterparts is rising. Microalgal carotenoid producers such as *Dunaliella salina* for β−carotene and *Haematococcus pluvialis* for astaxanthin are used in the industry. Food is one of the key sectors for carotenoid production, and estimates suggest that this industry will reach 2 billion USD worldwide in 2026 [85]. In Table 1, we have presented examples of algal metabolites with their health-promoting effects.

## 3. The Influence of Stress Conditions on Microalgal Cells

Initially, the main interest in the cultivation of microalgae was obtaining fatty acids for biofuel production. However, further research has proven that under stress conditions, microalgae accumulate valuable secondary metabolites, which can elevate the value of those organisms even more than algal lipids used for biofuel. The main factors that modulate the production of bioactive substances are abiotic stressors, which include light, nitrogen or phosphorus depletion, salinity, and xenobiotics, such as phytohormones [87].

### 3.1. Light

Light intensity, photoperiod, and light wavelength play a significant role during algae cultivation. In open cultivation systems, there is little to no control over natural lighting, which is influenced by geographical or seasonal changes. On the other hand, in closed photobioreactors, the conditions are controllable, and the risk of contamination is substantially lower. In microalgal cultivation, four light parameters are mainly considered: a light source (LEDs or fluorescent lamps could be selected), wavelength, light intensity, and photoperiod (which is a light-to-dark ratio) [88]. In *Dunaliella salina*, a halophilic species of microalgae and an efficient β-carotene producer, different light compositions affected the biosynthesis of valuable substances [89]. In their study, carotenoid production was induced by blue light irradiance, whereas biomass growth was accelerated by red light. Therefore, to optimize carotenoid production, the synergistic influence of two light wavelengths in a culture must be considered—to increase biomass productivity and improve accumulations of valuable pigments. A similar effect was shown by Kobayashi et al. [90]; in their experiment, blue light significantly induced carotenoid formation in *Haematococcus pluvialis*. After the intensive light stress, *H. pluvialis* becomes encysted, which is a resting state in which cells are surrounded by a thick wall and accumulate the red pigment—astaxanthin. Increased levels of this secondary carotenoid are considered a defense mechanism to scavenge reactive oxygen species. As proteomic analysis suggested, astaxanthin may be responsible for the majority of the antioxidant defense in *H. pluvialis* because the accumulation of this pigment led to the downregulation of SOD, catalase, and peroxidase enzymes [91]. Yusof et al. [92] have examined the influence of photoperiod on cell density and quantity of cellular compounds in three microalgal cultures (*Chlorella vulgaris, Isochrysis galbana, and Tetraselmis chuii*) and found that under the continuous light regime, the cell densities of all microalgae species were higher than during the photoperiod of 12:12 h. Moreover, a significant increase in the number of cells was observed in *C. vulgaris* and *T. chuii*. Continuous lighting has also caused an increase in chlorophyll and carotenoid levels in all tested microalgae. The authors suggest that pigments, as well as ascorbic acid and α-tocopherol, which concentrations also increased, play a complementary role in the reduction of oxidative stress. However, not all green algae need constant illumination to induce the biosynthesis of valuable metabolites. Krzemińska et al. [93] have shown that from the tested algal species, *Botrycoccus braunii* and *Scenedesmus obliquus* intensified the growth under continuous light, while three *Neochloris* species needed only the 12:12 photoperiod. Moreover, Wahidin et al. [94] have assessed the growth and lipid content of microalgae *Nannochloropsis* sp. grown under different light intensities and photoperiods. They have demonstrated that light intensity also has an important role in the growth of microalgae. Of the three evaluated light regimes (12:12; 18:6; 24:0), the continuous light was proven to be the best at the lowest light intensity (50 μmol m^−2^ s^−1^), and after 9 days of culture, the 12:12 illumination cycle had influenced the growth to the greatest extent for the highest light intensity (200 μmol m^−2^ s^−1^). However, the maximum specific growth rate and cell density for *Nannochloropsis* sp. was reached at the light intensity of (100 μmol m^−2^ s^−1^) and photoperiod of 18:06 (light:dark ratio). Varshney et al. [95] have shown the correlation between the light intensity, temperature, and the specific growth rate of two algae: *Asterarcys quadricellulare* and *Chlorella sorokiniana*. The maximum growth rate was reached at 200 μmol photons m^−2^ s^−1^, and in the temperature range of 23.5 to 43 degrees Celsius, the optimal temperature for the specific growth of both algal cultures was proven to be 37 °C. It is suggested that light causes the increase in total carotenoid concentration by the upregulation of phytoene synthase (PSY), which is a rate-limiting enzyme in carotenoid biosynthesis. Light directly causes the rise in Long Hypocotyl 5 (HY5) transcription factor levels, and HY5 induces further the expression of the phytoene synthase gene (*PSY*). In the darkness, Phytochrome Interacting Factor 1 (PIF1) plays an antagonistic role to HY5 and causes the decrease in PSY levels [96,97]. The PIF/HY5 mechanism also plays a role in temperature signaling. In *Arabidopsis thaliana*, PIFs are functioning at warmer temperatures, and they regulate pigment levels. In contrast, HY5 operates at lower temperatures. In the dark, the temperature does not have a significant impact on HY5. However, while the plant is exposed to red light, HY5 transcripts and protein levels increase and are higher in colder temperatures. HY5 in cold temperatures promotes the accumulation of anthocyanins and the production of ROS [96].

### 3.2. Salinity

The studies have also examined the influence of salinity stress on the growth and productivity of microalgae. Elloumi et al. [98] have carried out a study to evaluate the effect of salinity on the growth and productivity of *Scenedesmus* sp. microalgae, where they used varying concentrations of NaCl in an MDM medium. They have found that the high level of salinity inhibits the growth of microalgae, but the lower concentrations have promoted the growth of *Scenedesmus* sp. Furthermore, the levels of chlorophylls and carotenoids were elevated under low-concentration salt stress. Research carried out by Paliwal et al. [99] proves that the mild salinity stress (medium salinity increased to 0.2 M NaCl) improved carotenoid accumulation by 21% in microalgae *Synechocystis* sp. An increased salinity steadily decreased the contents of carotenoids, productivity, and growth of biomass (even from 0.2 M NaCl). However, the β-carotene content was improved significantly with the increase of salinity, and it has reached the peak concentration at the highest salinity level, at 1 M of NaCl. Schmidt et al. [100] have tested the metabolite content in the red alga *Pterocladiella capillacea* under cadmium stress and different salinities. They observed that the content of phenolic compounds was overall decreased, and the carotenoid content was elevated under Cu treatment and different salinities. Treatment with these two factors led to different profiles; almost all carotenoid levels have increased. However, increasing only the salinity significantly increased the concentration of carotenoids. BenMoussa-Dahmen et al. [101] have similarly demonstrated that the growth of *Dunaliella* sp. and *Amphora subtropica* was elevated under 3 M NaCl and 1M NaCl, respectively, and decreased below and above these optimal salt concentrations, which may suggest that salinity plays a major role in microalgal growth, and is even required for growth of the halophilic species such as *Dunaliella* sp. and *A. subtropica*. Furthermore, increased salinity improved pigment levels. While increasing salinity beyond the optimal growth conditions, they have demonstrated that the levels of chlorophylls decreased, but the total levels of carotenoids were significantly increased. On the other hand, Janchot et al. [102] demonstrated that potassium chloride stress caused a decrease in chlorophyll content in alga *Chlorococcum* sp., and the total levels of carotenoids have not increased significantly. However, KCl stress modulated and increased the levels of astaxanthin and cantaxanthin. The authors suggest that under stress conditions, *Chlorococcum* sp. might have shifted the biosynthesis to produce compounds from the β-carotene family.

### 3.3. Nutrients Deprivation

It is known that nutrient availability during the culture process, such as the limitation of nitrogen, can influence the growth of biomass and the production of algal metabolites. Ramos et al. [103] have shown that *Dunaliella salina* more intensively accumulates β-carotene under nutrient (nitrate) depletion. Furthermore, when the concentration of nitrate decreased in the control culture, the authors observed a rise in β-carotene accumulation. The highest accumulation of this pigment was observed under the combination of salt stress and nutrient limitation. In contrast, Coulombier et al. [104] have explored the impact of nitrogen deprivation on antioxidant properties and pigment accumulation in the green alga *Nephroselmis* sp., which possesses naturally high carotenoid content. During the exponential growth phase, the pigment contents have increased, but after nitrate depletion, the concentration of carotenoids and chlorophylls has decreased. The measured antioxidant activity was more than two times lower in the nitrogen-limited and starved cultures compared to the study control cultures. Even after nitrogen resupply, antioxidant activity improved, but it has not reached the earlier maximum antioxidant capacity. According to the author, all identified carotenoids contributed to the antioxidant capacity of the culture, mainly siphonoxanthin, neoxanthin, violaxanthin, antheraxanthin, and zeaxanthin. Forján et al., [105] conducted a study in which they tested the influence of the limitation of nitrogen, sulfur, and phosphorus on the carotenoid accumulation in *Nannochloropsis gaditana* microalga. The growth of the microalga was accelerated by delivering 5% CO_2_ instead of air alone or HCO_3_^−^ to the culture. On the contrary, deprivation of nitrate, sulfate, and phosphate has decreased both the growth rate and total carotenoids. After identifying and quantifying the content of individual carotenoids, researchers have found that the limitation of phosphate and sulfur led to an increase in the concentration of zeaxanthin and less of violaxanthin. 

### 3.4. Xenobiotics

Phytohormones are another factor that influences the growth of microalgae and the accumulation of pigments. In their literature review, Wang et al. [106] point out that phytohormones were researched for the regulation in land plants, but there are few studies on their effect and role in microalgae. However, their function may be similar in both algae and land plants. As an example: auxins, such as indoleacetic acid, can be found in microalgal cells. They are factors that are used during the growth and metabolism of algae. Salicylic acid is a phenolic phytohormone whose role has been reported in reaction to biotic and abiotic stresses, such as pathogen attacks, salinity, and UV radiation. Salicylic acid (SA) was reported to improve the concentration of pigments in *D. salina* to a greater extent than nitrogen deficiency (ND) or using both phytohormone and nitrogen limitation treatments. While ND and SA combined and ND alone increased the fresh weight more, compared to the control and even the treatment with SA [107]. Kozlova et al. [108] have tested the effect of epibrassinolide, brassinolide, indoleacetic acid, and abscisic acid, using their concentrations that corresponded to their amount in hydroponic wastewater, on the growth and productivity of the green alga *Scenedesmus caudata*. Phytohormones significantly increased biomass growth and cell size (except for abscisic acid in high concentrations). Furthermore, plant hormones have intensely improved carotenogenesis. Epibrassinolide has improved carotenoid accumulation by up to four times compared to the control group. Mousavi et al. [109] have shown that indoleacetic acid, benzyl amino purine, gibberellic acid, kinetin, salicylic acid, and γ-dimethylallyl aminopurine have increased the number of algae cell numbers, compared to the control group, while kinetin and indoleacetic acid had the greatest effect on the growth. Similar results could be observed in the accumulation of β-carotene, where all hormones had a positive effect on the concentration of this carotenoid in the cell, but kinetin and indoleacetic acid had the greatest influence on the total β-carotene content. In some cases, the combined treatment of phytohormones and other stressors may lead to different results. Kováčik et al. [110] tested the combined effect of copper (Cu) and salicylic acid (both 25 μM) on phenolic compounds and free amino acids in the *Scenedesmus quadricauda* green algae. The authors have discovered that copper induced an increase in arginine, histidine, methionine, and proline levels of the 17 free amino acids identified, while salicylic acid has substantially increased nearly all amino acids detected, except for a small increase in methionine levels. Compared to the SA treatment only, SA + Cu usage have increased further the levels of methionine. Cu decreased the number of soluble phenols, and SA had no effect, nor has it prevented the decrease of soluble phenols levels under Cu stress. Nevertheless, Cu, SA, and combined treatments have stimulated the increase of detected phenolic acids. The total sum of phenolic acids increased the most in SA and SA + Cu cultures. Authors suggest that the detected protocatechuic acid, which was increased under SA and SA + Cu stress, has high chelating strength, so it can provide a function in copper-related heavy metal stress. Moreover, Szpyrka et al. [111] have treated microalga *Planktochlorella nurekis* with two natural products—colchicine and cytochalasin B—to inhibit karyokinesis and cytokinesis within cells. The use of this approach has caused the rise in growth rate, cell size, and in DNA levels. Additionally, total lipids content increased by about 10 to 60 percent in all modified clones. Furthermore, the concentration of carotenoids was increased compared to the untreated cells.

## 4. Conclusions

Algae are unicellular or multicellular photosynthetic organisms that live in diverse environments and range in size from macro- to microalgae. Currently, the aspect of the production of valuable metabolites by microalgae is gaining popularity in biological and biotechnological research worldwide. These organisms are a potent source of lipids, pigments, carbohydrates, enzymes, and phenolic compounds. Under stress conditions induced by intensive or continuous light, nitrogen or phosphorus limitation, xenobiotics, salinity, and temperature, microalgae can overproduce valuable metabolites. Fatty acids are important algal metabolites that are used by the industry to produce biofuels. Although, the production of nutraceuticals, such as carotenoids and phenolic compounds, may have even more worth, especially in times of decreasing usage of combustion engines and considering the beneficial bioactive effects that secondary algal metabolites possess. However, the impact of environmental factors on the biosynthesis of carotenoids and phenolic compounds needs to be explored to a greater extent while comparing generated genomic transcriptomic, proteomic, metabolomic, and biochemical data. Modern techniques, such as liquid chromatography coupled with mass spectrometry and 2D electrophoresis, may be used to study the biosynthesis of valuable secondary metabolites in algae. The research can further expand our knowledge about the molecular pathways of valuable compound synthesis and contribute to new and improved applications for bioproducts derived from microalgae.

## Figures and Tables

**Table 1:** Examples of metabolites extracted from algae and their health-promoting effects.

Metabolite.	Genera/Species	Effects on Health	References
Gallic acid	*Spirogyra* sp.	Protects against UVB-induced oxidative stress and apoptosis. Vasorelaxant activity, protects against hypertension.	[42,43]
Phenolic extracts (Pyrogallol)	*Spirulina platensis*	Antioxidant, anti-inflammatory, hepatoprotective activities	[45]
Phytoene	*Dunaliella salina*	Anti-inflammatory activity, Inhibition of AGE accumulation, anti-aging effect	[79]
β-carotene	*Dunaliella salina*	Antiproliferative activity (cancer)	[82]
Lutein	*Chlorella ellipsoidea*	Prevention and treatment of type-2 diabetes	[62]
Astaxanthin	*Haematococcus pluvialis*	Antioxidant, anti-inflammatory, anti-apoptotic activities	[81]
Zeaxanthin	*Chlorella ellipsoidea*	Prevention and treatment of type-2 diabetes, protection from cardiac disfunction	[62]
Fucoxanthin	*Undaria pinnatifida (brown alga), Codium fragile, Phaeodactylum tricornutum*, *Cyclotella cryptica*	Anti-angiogenic activity, Inhibition of AGE accumulation	[77,78]
Siphonoxanthin	*Undaria pinnatifida (brown alga), Codium fragile*	Anti-angiogenic activity	[77,86]

## Data Availability

Not applicable.

## Abbreviations

AGEs	Advanced glycation end products
Bcmo1	β-carotene oxygenase 1
CAT	
Catalase	
C/EBPα	CCAAT enhancer-binding protein alpha
cas3	Caspase 3
CCD4	Carotenoid cleavage dioxygenase 4
*D*-GAL	D-galactose
DW	
Dry weight	
FGF-2	Fibroblast growth factor 2
GAE	
Gallic acid equivalent	
GLUT-4	Glucose transporter 4
GPx	Glutathione peroxidase
IL-1β/6/10	Interleukin 1 beta/6/10
iNOS	
Inducible nitric oxide synthetase	
ITS2	Internal transcribed spacer 2
PPARγ	Peroxisome proliferator-activated receptor gamma
QE	Quercetin equivalent
RAR- α	Retinoid receptor alpha
ROS	Reactive oxygen species
rbcL	Ribulose-bisphosphate carboxylase
SIRT3/6	Sirtuin 3/6
SOD	Superoxide dismutase
TNF-α	Tumor necrosis factor alpha
UCP1/2	Uncoupling protein 1/2
